# β-Selinene-Rich Essential Oils from the Parts of *Callicarpa macrophylla* and Their Antioxidant and Pharmacological Activities

**DOI:** 10.3390/medicines4030052

**Published:** 2017-07-10

**Authors:** Mahesh Chandra, Om Prakash, Ravendra Kumar, Rakesh Kumar Bachheti, Brij Bhushan, Mahesh Kumar, Anil Kumar Pant

**Affiliations:** 1 Department of Chemistry, Graphic Era University, Dehradun, pin code248 002, Uttarakhand, India; mahesh2747@gmail.com (M.C.); rkbachheti@gmail.com (R.K.B.); brijbushaniitr@gmail.com (B.B.); 2 Department of Chemistry, College of Basic Sciences and Humanities, G.B. Pant University of Agriculture and Technology, Pantnagar, U.S. Nagar, pin code 263 145 Uttarakhand, India; ravichemistry.kumar@gmail.com (R.K.); anilpant54@gmail.com (A.K.P.); 3 Department of Medicine, College of Veterinary Sciences, G.B. Pant University of Agriculture and Technology, Pantnagar, U.S. Nagar, pin code 263 145 Uttarakhand, India; maheshkumar_epm@rediffmail.com

**Keywords:** β-selinene, phylocladene, pharmacology, *Callicarpa macropylla*, caryophillene oxide

## Abstract

**Background:** *Callicarpa macrophylla* (Varbenaceae) is a medicinal shrub and is traditionally used in India, China, and South Asia. **Methods:** The plant material was collected from lower Himalayan region of Uttarakhand in India. The essential oils from three different aerial parts were analyzed by GC-MS. Antioxidant activity, phenolic assay, and various pharmacological activities were determined by using existing methods which are generally practiced widely. **Results:** Over 51, 53, and 40 compounds were identified in *C. macrophylla* leaves essential oil (CMLEO), *C. macrophylla* pre mature seeds and fruits essential oil (CMEO-I) and *C.macrophylla* mature seeds and fruits essential oil (CMEO-II), respectively. These oils differ in relative contents of major compounds viz; β-selinene (37.51% in CMLEO, 44.66% in CMEO-I and 57.01% in CMEO-II), phyllocladene (9.76% in CMLEO, 5.80% in CMEO-I and 12.38% in CMEO-II), caryophelline oxide (7.34% in CMLEO, 8.74% in CMEO-I and 5.0% in CMEO-II), 9E-epi-caryophelline (6.23% in CMLEO, 1.27% in CMEO-I and 3.43% in CMEO-II), longipinocarvone (4.96% in CMLEO, 1.17% in CMEO-I and 2.0% in CMEO-II), and 1,8-cineole (2.23% in CMLEO, 3.10% in CMEO-I and 1.62% in CMEO-II). The oils exhibited good in vitro antioxidant activity. The maximum activity was found in CMEO-II with IC_50_ values 7.37 ± 0.11, 11.49 ± 0.87, 14.59 ± 0.18, 15.66 ± 0.03, and 17.49 ± 0.13 µL/mL. The essential oils showed qualitative and quantitative diversity in the makeup of essential oils constituents. The oils were found to exhibit anti-inflammatory, analgesic, and antipyretic activity on swiss albino mice compared to the standard drugs, viz; ibuprofen, paracetamol and indomethacin. **Conclusion:** It is inferred from the study that the plant parts can be used scientifically in traditional systems as folk herbal medicine. Furthermore, we have generated a database for future reference and judicious exploitation of these oils from their natural setting.

## 1. Introduction

Many plants have been found as a source of natural antioxidants and base components for new drug formation from their essential oils. The Indian Himalayan region is a large repository of medicinal and aromatic plants. In present study, *C. macrophylla* growing wildly in Uttarakhand Himalaya of India was investigated for chemical composition and pharmacological activity of its essential oils. This plant belonging to family Varbenaceae is an erect, stellate-pubescent, perennial shrub, with stems terete and leaves in sub or unequal pairs on 0.3–1.5 cm long petioles, elliptic-dentate with gland- tipped teeth, glabrescent or so above, 10–20 (–25) × 4–6 (–8) cm while flowers in axillary, 1–3 cm long, peduncled, cymose, corymbose panicles [1]. In this plant, 20 out of 40 species have been reported for their ethno-medical uses. Several members in Chinese and South Asian tradition have been reported as antibiosis, antiphlogosis, and hemostasis drugs [2]. The parts of *C. macrophylla* have been used to treat many ailments like rheumatism and stomach problems. The bark is used to cure cuts and injuries [3]. As an herbal folk medicine, the seeds and roots of this shrub are used for digestive and abdominal troubles in India [4]. 16α, 17-isopropylidene-3-oxophyllocladane (iso-propylidinocalliterpenone) along with calliterpenone and its monoacetate have been reported in methanol extracts from the residual water extracts left after distillation of the essential oil [5]. Analgesic, anti-pyretic, and anti-inflammatory activities have been reported in aqueous and ethanolic extracts of this plant [6,7]. Terpenoids viz; spathulenol (18.1%), germacrene B (13.0%), bicyclogermacrene (11.0%), globulol (3.3%), viridiflorol (2.6%), a-guaiene (2.3%), and g-elemene (2.0%) have been reported in the essential oil of *C. japonica* with different chemical makeup from another species, *C. americana,* growing in Mississippi [8]. We have also reported anti-inflammatory and analgesic activities in aqua-alcoholic leaf extracts of *C. macrophylla* [9].

Tetracyclic diterpenes, calliterpenone, and calliterpenone-monoacetate from the petroleum ether extract, along with β-salinene (41.6–29%) and α-salinene (6–1.7%) in volatile oil of the aerial parts of *C. macrophylla* have been reported [10,11]. In view of its medicinal uses in traditional systems, in the present investigation of different parts of the plant (leaves, pre-mature and mature seeds and fruits) were taken to isolate essential oils and examine the chemo-diversity, antioxidant, and pharmacological activities of this important shrub.

## 2. Materials and Methods

### 2.1. Plant Material

Fresh aerial parts from *C. macrophylla* were collected during September, October 2011. The plant was taxonomically authenticated by Dr. D.S. Rawat (Assistant Professor & Plant Taxonomist), Department of Biological Science, G.B. Pant University of Agriculture & Technology, Pantnagar. The Herbarium specimen was preserved and deposited in the department.

### 2.2. Isolation of Essential Oil

The crushed plant parts were hydro-distilled using Clevenger’s type apparatus for over 8 h. The essential oils were extracted by diethyl ether and dried by adding anhydrous Na_2_SO_4_. Removal of solvent yielded 0.20% (*w/v* both in leaves and pre-mature seeds & fruit) and 0.15% (*v/w* in mature seeds & fruits) of essential oils, respectively. 

### 2.3. GC Analysis 

Gas chromatographic analysis was done on a Nucon-GC 5765 system interfaced with a flame ionization detector(FID) and capillary colum (DB-5, 30 m × 0.32 i.d.). The column temperature was induced at 60 °C for the first 5 min and then programmed with the RAM of 3 °C/min up to 210 °C, and finally isothermally for 10 min. Detector temperature was 210 °C and N_2_ was used as carrier gas (flow rate 50 kg/cm^2^). The injection volume of essential oil used was 0.1 µL. The percent composition of oil constituents was determined with the help of a FID.

### 2.4. GC/MS Analysis 

The essential oils were analyzed using a Shimadzu GC-2010 interfaced, with a Shimadzu GCMS-QP2010 Plus mass selective detector, having omega wax column (30 m × 0.25 mm, 0.25 μm). The column initial oven temperature was 60 °C and then programmed at 3 °C/min to final oven temperature 240 °C with isothermal for 20 min. The injector temperature was 270 °C. Carrier gas used was helium with a flow rate of 2.42 mL/min and split ratio of 40:1 For MS detection; electron ionization (70 eV) was used as ionization technique. The ion source temperature was 230 °C with 280 °C as the interface temperature. The components were identified by their relative retention times and matching mass spectra with those of standards (main components), from NIST/Wiley library, data of the main system and those published in the literature [12].

### 2.5. Antioxidant Assay

#### 2.5.1. Reducing Power Activity

The reducing power was evaluated by the procedures reported earlier [13]. In brief 5, 10, 15, 20, and 25 µL of essential oils (EOs) were added in 2.5 mL of phosphate buffer (200 mM, pH 6.6) and 2.5 mL of 1% K_3_[FeCN_6_], followed by 2.5 mL of 10% Cl_3_CCOOH after incubation at 50 °C for 20 min. The content was centrifuged for 10 min at 650 rpm. 5 mL of the supernatant was mixed with 5 mL of distilled water and 1 mL of 0.1% FeCl_3_. The optical density (OD) of the solutions was measured at 700 nm using UV-spectrophotometer (Thermo scientific, Waltham, MA, USA). The same procedure was followed for controls and standards. BHT, catechin, and gallic acid were used as standard antioxidants. 

#### 2.5.2. Effect on the Chelating Activity of Fe^2+^

The procedure is based on the Fe^2+^ chelating ability of the antioxidant measured calorimetrically at 562 nm using ferrous ion-ferrozine as a reference complex [14]. 0.1 mL of 2 mM FeCl_2_·4H_2_O and 0.2 mL of 5mM ferrozine were added to 5–25 μL of essential oils followed by methanol to make up the volume to 5 mL. The solutions were homogenized and allowed to react for 10 min. OD was measured at 562 nm. The chelating activity on Fe^2+^ of EOs were compared with that of EDTA (0.01 mM) and citric acid (0.025 M). The following equation was used to calculate the percent of chelating activity.

% chelating activity = [1 − A_t_/A_0_] × 100 (1)

(A_t_ = absorbance of the sample, A_0_ = absorbance of the control at 562 nm).

#### 2.5.3. DPPH Radical Scavenging Activity

The scavenging effect on the DPPH radical was evaluated by following the developed and reported procedures [15]. 5, 10, 15, 20, and 25 µL of EOs were mixed with 5 mL of 0.004% freshly prepared DPPH solution in CH_3_OH. The solutions were placed in the dark for 30 min. OD of the samples, standards, and control were read at 517 nm. BHT, catechin, and gallic acid were used as standards. The percent DPPH radical scavenging activity was calculated by the following equation.

% DPPH radical scavenging activity = [1 − A_t_/A_0_] × 100 (2)

(A_t_ = absorbance of the sample, A_0_ = absorbance of the control at 517 nm).

#### 2.5.4. NO Radical Scavenging Activity

Nitric oxide (NO) obtained from sodium nitroprusside (SNP) was measured by the Griess reagent with the composition of 1% sulfanilamide, 0.1% naphthylethylenediamine dichloride, and 2.0 mL orthophosphoric acid. The scavengers of NO competed with oxygen, leading to reduced production of NO. 2 mL of SNP (10 mM) in phosphate buffer saline (PBS) with pH 7.4 was homogenized with different concentrations (5–25 µL/mL) of EOs dissolved in acetone. The content was incubated at 25 °C for about 150 min. OD of the pink color developed was read at 546 nm. Ascorbic acid was taken as standard [16]. NO scavenging activity was calculated as:

% NO scavenging = [1 −A_t_/A_0_] × 100 (3)

(A_t_ = absorbance of sample, A_0_ = absorbance of control at 546 nm).

#### 2.5.5. Super Oxide Radical Scavenging Activity

In brief 1 mL nitroblueterazolium (156 mM), 1 mL nicotinamide adenine dinucleotide (468 mM) and 0.1 mL of phenanzinemethosulphate solution (PMS) in 0.1 M of phosphate buffer solution (pH 7.4) was mixed to various concentrations (5–25 μL) of EOs followed by incubation at 25 °C for 5 min. The OD was measured at 560 nm against blank containing all reagents except PMS. Ascorbic acid was taken as standard [17]. The % super oxide radical scavenging activity was calculated as:

% superoxide radical scavenging = [1 −A_t_/A_0_] × 100 (4)

(A_t_ = absorbance of sample, A_0_ = absorbance of control at 560 nm).

#### 2.5.6. OH Radical Scavenging Activity

The hydroxyl (OH) scavenging activities of EOs were evaluated by using the developed protocols [17]. Briefly 60 μL FeSO_4_.7H_2_0 (1 mM), 90 μL aq. 1, 10 phenanthrolein monohydrate (1 mM), 2.4 μL (0.2 M) phosphate buffer (pH 7.8) and 150 μL of H_2_O_2_ (0.17 mM) was added in 1.5 mL of EOs (5–25 µL). OD was measured at 560 nm for samples control and standards (ascorbic acid). The % OH scavenging activity was calculated as:

% OH scavenging = [1 −A_0_/A_t_] × 100 (5)

(A_t_ = absorbance of sample, A_0_ = absorbance of the control at 560 nm). 

### 2.6. Evaluation of Pharmacological Activities 

Permission from the institutional ethical committee (Registration No. 330/CPCSEA, Dated, 1 Marth 2001, however the experiments were conducted in the month of December 2012) was taken prior to execute the experiments. Swiss albino mice were procured from the Lab Animal Division of the Central Drug Research Institute, Lucknow, U.P. in India. The animals were randomly divided into eleven groups with six mice in each and were kept under standard laboratory conditions. Three concentrations (5%, 10%, and 20%) of EOs were given orally with the dose level of 10 mL/kg body weight. All the concentrations of EOs were separately triturated by addition of small amount of tween-20 and saline water to make the final volume of 10 mL. Ibuprofen, indomethacin, and paracetamol were used as standard drugs and saline water as control.

### 2.7. Anti-Inflammatory Activity 

#### 2.7.1. Carrageenan-Induced Paw Edema 

The anti-inflammatory activity of EOs were evaluated according to the protocols reported [18]. Briefly, edema was induced by injecting carrageenan (0.1 mL, 1% w/v in saline) in the sub plantar tissue of the right hind paw of mice. The ninth group was given EOs 5, 10, and 20% as 10 mL/kg body weight, group ten was given ibuprofen (40 mg/kg b. wt.), while group eleven received only saline water. The paw volumes were measured plethysmometrically at 1, 3, and 24 h after the carrageenan injection. It has been reported that formation of edema is a result of synergism between inflammatory mediators which provoke vascular permeability and mediate the blood flow [19]. The decrease in the paw volume in comparison to control was taken as anti-inflammatory effect.

#### 2.7.2. Formaldehyde-Induced Inflammatory Activity

This activity of the EOs were studied in HCHO induced arthritis. Briefly, 0.1 mL HCHO (1%) solution was injected in the right hind paw of the mice [20]. The EOs were administered orally every day in the morning during tenure (10 days) of experiments. Ibuprofen suspension (40 mg/kg b. wt.) was used as standard anti-inflammatory drug. The control group was given only saline water. Paw volumes of all the mice were measured plethysmometrically.

### 2.8. Analgesic Activity

#### Acetic Acid-Induced Abdominal Writhing Test 

In this activity, the animals were treated with glacial acetic acid intraperitoneally to induce pain sensation [21]. After 1 h, 0.2 mL of EOs, ibuprofen (40 mg/kg b. wt.), and saline water were orally administered. The numbers of writhings were counted for 30 min in each mouse. The reduction of writhing in mice by ibuprofen was compared and the percentage of pain protection was calculated using the following formula:

% writhing = (T/C) × 100; % Inhibition = (C − T/C) × 100(6)

T = treatments (group I–IX); C = Control saline group (X).

### 2.9. Antipyretic Activity 

To evaluate antipyretic activity pyrexia was induced in mice by subcutaneous injection of 20% brewer’s yeast *(Sacchromyces cerevisiae)* (10 mg/kg b. wt.) as per the reported protocol [22]. The mice were maintained in a quiet laboratory environment for 18 h in order to raise body temperature. At the 19th hour, the rectal temperature was recorded. After 18 h of injection of yeast, immediately essential oils (5%, 10% and 20%), and paracetamol (33 mg/kg b. wt.) were administered orally. The control group was given only 0.2 mL normal saline. The temperature was monitored at hourly intervals in all the mice up to 3 h and the percentage reduction in rectal temperature was calculated by considering the total fall in temperature to normal level as 100%.

% reduction = {(B − C/B − A) × 100}(7)

A = normal temperature B = Pyrexia temperature C = temp at hourly interval

### 2.10. Assessment of Toxicity

In order to evaluate the toxic effect of essential oils, different doses (40%, 60%, and 80%) at 10 mL/kg body weight were given to experimental animals. For toxic effect of drug under experiment, the behavioral changes were recorded for 24 h and the numbers of deaths if any were recorded up to 48 h.

### 2.11. Statistical Analysis 

The experimental data generated were represented as mean ± S.E and the results were analyzed using one way analysis of variance. The value of *p* < 0.05 were considered to be statistically significant.

## 3. Results and Discussion

Over 40–53 compounds were identified in CMLEO, CMEO-I, and CMEO-II which contributed to 96.55%, 94.56%, and 95.35% of the total oil, respectively. The constituents identified in CMLEO, were β-selinene (37.51%), phyllocladene (9.76%), caryophelline oxide (7.34%), caryophelline-9-epi (E) (6.23%), longipinocarvone (4.96%), β-caryophelline (3.26%), juniper camphor (3.13%), vulgarone (2.92%), 1, 8-cineole (2.23%), α–muurolol (1.76%), and sphathulenol (1.06%) (Table 1). In CMEO-I, the identified constituents β-selinene (44.66%), caryophelline oxide (8.74%), phyllocladene (5.80%), 1,8-cineole (3.10%), juniper camphor (3.03%), longicamphenylone (3.08%), aromadandrene (2.14%), sphathulenol (2.10%), caryophelline-9-epi(E) (1.27%), longipinocarvone (1.17%), β-pinene (1.07%), and vulgarone (1.02%) were major compounds along with other minor ones (Table 1). β-selinene (57.01%), phyllocladene (12.38%), caryophelline oxide (5.0%), 9E-epi-caryophelline (3.43%), β-pinene (2.32%) longipinocarvone (2.0%), 1,8-cineole (1.62%), β-caryophelline (1.84%), vulgarone(0.40%), sphathulenol (0.30%), and aromadandrene (0.19%) were identified in CMEO II (Table 1). 

β-Salinene has been found as a major compound in all the EOs. The oils also showed diversity both in qualitative and quantitative makeup of essential oils. CMEO-I contained longicamphnylone (3.08%), 4-camphenylbutan-2-one (0.80%) cedren-13-ol (0.52%), nopinone (0.50%), hexanoic acid (0.47%), terpinen-4-ol (0.43%), bornyl acetate (0.42%), and leden oxide-I (0.40%) which were absent in other oils. Muurolene-14-oxy-α (2.50%), α-muurolol (1.76%), β-copaen-4-α-ol (1.03%), globulol (0.42%), β-oplopenone (0.33%) etc were found only in CMLEO, while 5-octen-2-one (0.53%), β-ocimene (0.31%), α-pinene (0.40%), and khusinol (0.10%) were identified only in CMEO-II (Table 1). 

### 3.1. Antioxidant Assay 

#### 3.1.1. Reducing Power 

**Figure 1:**
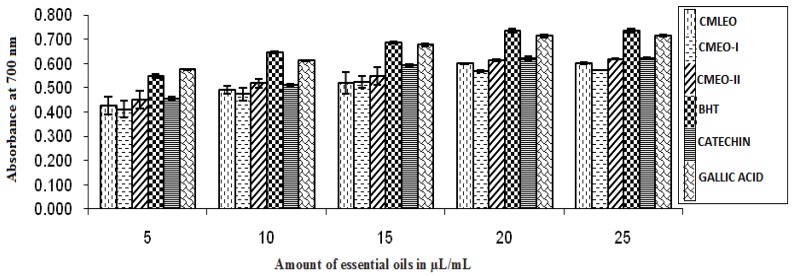
Reducing power activity.

The essential oils showed good reducing activity as the function of concentration compared to standard antioxidants (BHT, catechin, and gallic acid). CMEO-II showed the highest reducing property of the extracts (Figure 1).

#### 3.1.2. Ability of Chelating Fe^2+^ Ion

**Figure 2:**
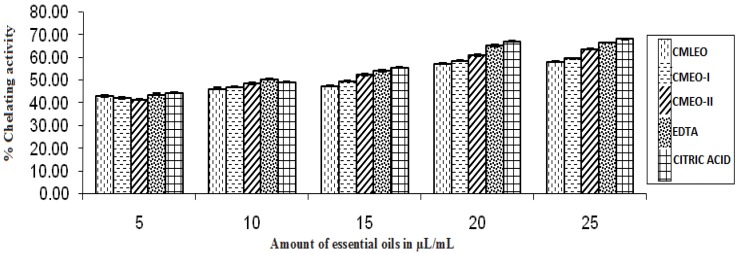
Chelating activity.

As shown in Figure 2, all the EOs revealed good Fe^2+^ chelating with maximum chelating values of 63.82% recorded for CMEO-II, at 25 µL/mL of dose level compared to standard antioxidants.

#### 3.1.3. DPPH Radical Scavenging Activity

**Figure 3:**
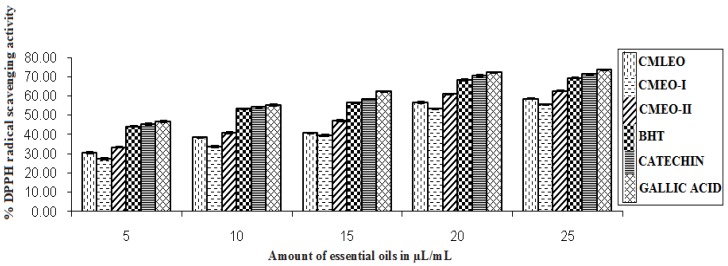
DPPH radical scavenging activity.

The EO showed good to moderate DPPH radical scavenging activity. CMEO-II was a better DPPH radical scavenger than CMLEO and CMEO-I, compared to standard antioxidant viz. BHT, catechin, and gallic acid (Figure 3). DPPH scavenging activity was observed between 55.67–62.63% at highest concentration (25 µL) among the oils. However, the standard showed slightly stronger activity in the range of 69.39–73.58%. 

#### 3.1.4. Superoxide Radical Scavenging Activity

**Figure 4:**
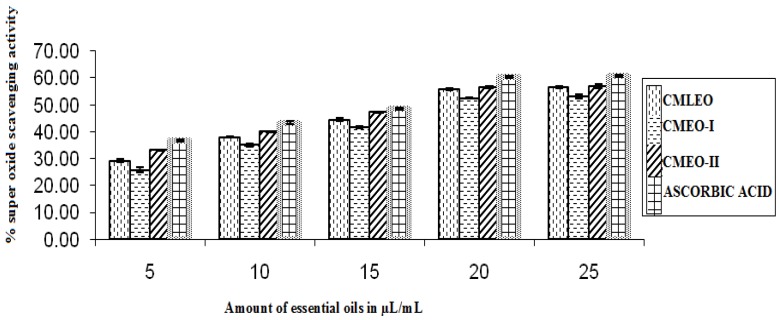
Super oxide scavenging activity.

CMEO-II, CMEO-I, and CMLEO exhibited good superoxide scavenging activity with maximum at higher concentration. CMLEO-II revealed highest scavenging activity (56.98 ± 0.630%) followed by CMLEO (56.49 ± 0.412%) and CMEO-I (53.13 ± 0.724%), respectively, as compared to ascorbic acid (60.76 ± 0.313%) (Figure 4).

#### 3.1.5. NO Radical Scavenging Activity 

**Figure 5:**
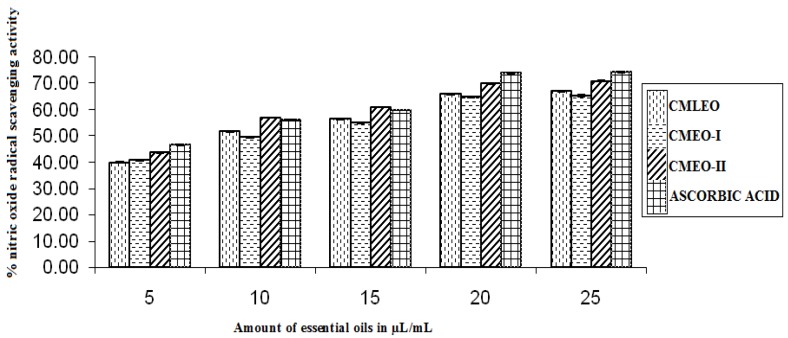
Nitric oxide radical scavenging activity.

The EOs were good NO radical scavengers but moderate in comparison to the standard, ascorbic acid. CMEO-II was the strongest NO scavenger among the oils and scavenged 43.51 ± 0.329–70.68 ± 0.115% NO radical from 5 µL to 25 µL (Figure 5).

#### 3.1.6. OH Radical Scavenging Activity

**Figure 6:**
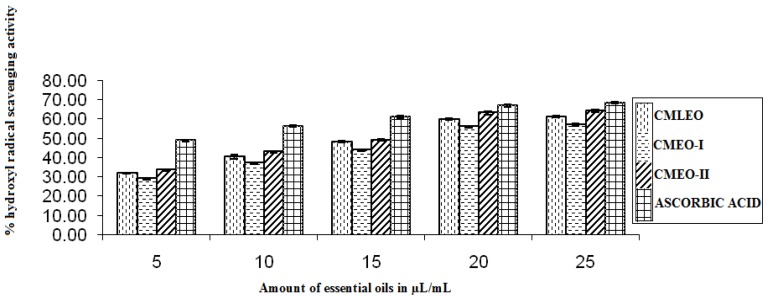
Hydroxyl radical scavenging activity.

The oils exhibited OH radical scavenging in a dose-dependent manner similar to DPPH, NO, and superoxide methods. Maximum scavenging activity was observed at the concentration level of 25 μL in ascorbic acid (68.48 ± 0.708%) followed by CMEO-II (64.4 ± 0.900%), CMLEO (61.22 ± 0.340%), and CMEO-I (57.03 ± 0.520%), respectively (Figure 6).

The results obtained as IC_50_ values for scavenging and chelating activity of the EOs indicated their efficiency against oxidative stress as well as free radicals. CMEO-II showed the minimum IC_50_ value in all the activity tests among the oils and the highest antioxidant activity, but was moderate in comparison to the standards (Table 2). The IC_50_ values for chelating activity were found in the order of EDTA (IC_50_ = 9.27 ± 0.11 µg/mL), followed by citric acid (IC_50_ = 9.42 ± 0.95 µg/mL), CMEO-II (IC_50_ =11.49 ± 0.87 µL/mL), CMEO-I (IC_50_ = 13.42 ± 0.17 µL/mL), and CMLEO (IC_50_ = 14.38 ± 0.27 µL/mL). The DPPH radical is a highly reactive species and used for evaluation of free radical scavenging activity of antioxidants. It accepts an electron and a hydrogen to become a stable diamagnetic molecule [18,19]. The scavenging effect of the EOs and standards with the DPPH radical were observed in the following order: gallic acid (IC_50_ = 7.95 ± 0.11 µg/mL) > catechin (IC_50_ = 8.18 ± 0.11 µg/mL) > BHT (8.55 ± 0.10 µg/mL) > CMEO-II (IC_50_ =15.66 ± 0.03 µL/mL) > CMLEO (IC_50_ =18.35 ± 0.18 µL/mL) > CMEO-I (IC_50_ = 20.19 ± 0.11 µL/mL). It has been reported earlier that the plant-derived products mitigate the harmful effects produced by nitric oxide radicals in human body [20,21]. In the present investigation, the level of nitric oxide was decreased with increased concentration of the EOs. CMEO-II had the strongest anti-radical activity with the IC_50_ value 7.37 ± 0.11 µL/mL, which was comparable to ascorbic acid (Table 2). It has been reported that nitric oxide production becomes high in tumor tissues, plasma, and other physiological and pathological processes [23]. The superoxide anion is a forerunner of reactive oxygen species and produces destructive effects to cellular systems [24]. Photochemically generated O_2_^.^ reduces to NBT and forms blue formazan [25]. It has been observed that inhibition of formation of the blue formazan is a function of the concentration of the essential oils, which were found to be efficient scavengers of superoxide radicals and their activity were moderate compared to ascorbic acid. Damage to the adjacent biomolecules by the most reactive hydroxyl radical has been reported along with oxidative damage to DNA, lipids, and proteins [26,27]. The present study reveals the maximum hydroxyl radical scavenging effect of essential oils at the concentration level of 25 µL/mL.

All the essential oils had low amounts of phenolic compounds but showed good antioxidant activity. The diversified mono- and sesquiterpenoids present in the complex mixture of essential oils might be responsible for the good antioxidant activity because of synergetic effects of the constituents. This can be evidenced by a report which says that antioxidant capacity is affected by other bioactive compounds and could involve synergistic effects [28].

### 3.2. Anti-Inflammatory Activity

#### 3.2.1. Mice Paw Edema (Carrageenan-Induced)

A gradual increase in carrageenan-induced hind paw edema volume of mice in the control and sample groups was observed. However, in CMEO-II, CMLEO, CMEO-I, and standard drug treated groups, a significant reduction in the volume of edema was observed (Table 3). Other concentrations of oils showed moderate activity in comparison to the standard drug. The inhibitory effect of the oils was observed to be the highest with a dose level of 20% oils at 24 h, as 21.16% in CMEO-II, 17.78% in CMLEO, and 16.30% in CMEO-I, whereas ibuprofen produced a 37.17% reduction in the same experimental conditions (Table 3).

#### 3.2.2. Formaldehyde-Induced Inflammatory Activity

CMEO-II, CMLEO, and CMEO-I resulted in moderate inhibition in paw volume compared to ibuprofen. CMEO-II revealed the greatest inhibition in paw volume (2.23 mm^3^ on day 1 and 2.20 mm^3^ on day 10), followed by CMLEO (2.3 mm^3^ on day 1 and 2.22 mm^3^ on day 10) and CMEO-I (2.26 mm^3^ on day 1 and 2.24 mm^3^ on day 10) at 20% dose level. The inhibition property was observed in a dose-dependent manner (Table 4).

### 3.3. Analgesic Activity

CMEO-II, CMLEO, and CMEO-I showed significant analgesic activity with 35.33%, 30.04%, and 25.72% inhibition at 20% dose level, respectively, compared to the standard drug, ibuprofen, which inhibited 43.99% (Table 5). Prostaglandins have been reported to enhance inflammation and pain by increasing capillary permeability [29]. An analgesic substance reduces the number of writhings due to pain. It has been reported that analgesic drugs inhibit the synthesis of prostaglandins [30]. It can be inferred that the EOs of *C. macophylla* are responsible for the reduction of prostaglandin synthesis and exhibit analgesic properties.

### 3.4. Antipyretic Activity

All the oils revealed significant antipyretic activity. Yeast significantly induced pyrexia by increasing rectal temperature 18 h after injection. The reduction in temperature was observed up to 3 h. A maximum reduction in pyrexia of 1.05 °C (96.33%) was observed in paracetamol-treated group up to 24 h. The uniform fall in temperature up to 24 h was observed by increasing the dose (Table 6). It has been reported in previous studies that the lipid per oxidation process is enhanced by increasing body temperature [31]. 

Infection in the body is a cause of fever or other diseased states like tissue damage, inflammation, and graft rejection etc. [32]. Regulation of body temperature requires equilibrium between the production and dissipation of heat. The present study exhibited significant antipyretic properties of EOs on brewer’s yeast-induced pyrexia in mice. The antipyretic activity of EOs may be due to interference on the biosynthesis of prostaglandins. The result of this study revealed that *C. macrophylla* can be beneficial to treat inflammations, pain, and fever. These activities might be due to presence of a complex mixture of terpenoids.

The essential oil of *C. macrophylla* showed activities in various degrees against inflammation, pain, and fever. According to the research, higher levels of prostaglandin, particularly PGE2, produce inflammation, pain, and fever because of cyclooxygenase activation [33]. As a result, we suppose that some active constituents of the essential oils could inhibit cyclooxygenase activity. It has been reported that the carrageenan-induced paw edema in rats is susceptible to cyclooxygenase (COX) inhibitors. These have been used as non-steroidal anti-inflammatory agents and are also suitable for assessing the anti-oedematous effects of natural products [34,35].

### 3.5. Acute Toxicity

Essential oils from parts of *C. macrophylla* neither cause any changes in behavior nor any death of mice under experimentation during the period of observation. It was thus considered that the EOs were practically non-toxic.

## 4. Conclusions

The EOs from *C. macrophylla* showed a qualitative and quantitative make-up of constituents. The oils also showed different pharmacological activities. From the results, it could be inferred that the essential oils might be used as drugs for treatment of inflammation, fever, and pain in traditional systems of medicine with scientific knowledge. Clinically, this herb can be a good source of herbal medicine for the treatment of diseases indigenously. The study will also help to generate a database of species which can be exploited scientifically and judiciously in the future by local people and so that ecological balance is maintained. 

## Figures and Tables

**Table 1:** Essential oil composition from different parts of *Callicarpa macropylla*.

S.N.	Compounds Name	KI/RI	FID %		
			CMLEO	CMOE-I	CMEO-II
1	hex-2*E*-enal	850	0.20	-	-
2	α-pinene	933	0.06	-	0.40
3	β-pinene	943	0.53	1.07	2.32
4	1-octene-3one	943	0.02	0.12	0.02
5	3-octanone	952	0.10	0.12	0.06
6	banzaldehide	960	0.14	-	-
7	sabinene	972	0.17	0.40	0.25
8	hexanoic acid	979	-	0.47	-
9	myrcene	991	-	-	0.04
10	hex-3*Z*-ethyl acetate	1008	0.01	-	-
11	p-cymene	1025	0.13	0.41	0.14
12	limonene	1030	-	0.15	0.12
13	1,8-cineole	1032	2.23	3.10	1.62
14	β-ocimene	1046	-	-	0.31
15	2-nonanone	1052	0.02	-	-
16	*trans*-2-octenal	1067	-	0.27	-
17	*cis* linalool oxide	1069	-	0.21	-
18	*trans* linalool oxide	1086	-	0.15	-
19	linalool	1101	0.20	0.86	-
20	nopinone	1139	-	0.50	-
21	sabina ketone	1154	0.03	-	-
22	pinocarvone	1164	-	0.64	0.45
23	terpinen-4-ol	1180	-	0.43	-
24	myrtenal	1197	0.23	0.74	0.20
25	1-butyryl-1,2,3,6-tetrahydropyridine	1249	0.05		
26	3,9-dodecadiyn	1249	0.37	-	-
27	bornyl acetate	1285	-	0.42	-
28	leden oxide (I)	1293	-	0.40	-
29	myrtenal acetate	1326	-	0.19	-
30	α-copaene	1375	0.35	-	0.27
31	β-elemene	1390	0.98	0.82	0.61
32	β-cubebene	1392	0.10	0.09	0.48
33	α-gurjunene	1406	0.50	0.95	0.15
34	nopyl acetate	1413	-	0.26	-
35	β-caryophelline	1424	3.26	-	1.84
36	(*E*) caryophellene	1424	0.44	-	0.11
37	aromadandrene	1438	-	2.14	0.19
38	4-camphenylbutan-2-one	1451	-	0.80	-
39	α-humulene	1454	-	-	0.14
40	aromadendrene oxide II	1462	0.43	0.46	0.25
41	9*E*-epi-caryophelline	1464	6.23	1.27	3.43
42	α-selinene	1474	0.26		
43	α-cubebene	1480	0.11	-	-
44	ar-curcumene	1480	0.19	0.10	0.14
45	β-selinene	1492	37.51	44.66	57.01
46	amorphene	1502	0.41	-	-
47	perhydropyrene	1502		0.37	
48	caryophelline oxide	1507	7.34	8.74	5.0
49	δ-cadinine	1518	0.85	0.41	0.59
50	*trans*-calamene	1527	0.35	0.15	0.31
51	globulol	1530	0.42	-	-
52	Z-α-bisaboline epoxide	1531		0.21	
53	α-agarofuron	1548	-	0.40	-
54	(*E*)-nerolidol	1561	-	0.20	-
55	longicamphenylone	1563	-	3.08	-
56	longipinocarvone	1569	4.96	1.17	2.0
57	sphathulenol	1576	1.06	2.10	0.30
58	β-copaen-4 α-ol	1590	1.03	-	-
59	*trans* longipinocarveol	1590	0.63	0.71	-
60	fokienol	1596	0.38	0.31	-
61	salvial-4 (14)-en-1-one	1596	0.73	-	-
62	β-oplopanone	1607	0.33		
63	humulene epoxide II	1613	0.21	-	-
64	*Z*-3-hexadecane-7-yne	1637	-	0.31	-
65	solavetivone	1645	0.99	0.30	0.40
66	cedren-13-ol	1646	-	0.52	-
67	vulgarone	1649	2.92	1.02	0.40
68	α-muurolol	1651	1.76	-	-
69	cadalene	1677	-	0.32	-
70	khusinol	1679	-	-	0.10
71	juniper camphor	1696	3.13	3.03	-
72	*cis*-lanceol	1760	0.12	-	-
73	14-oxy α-muurolene	1767	2.50	-	-
74	phyllocladene	1789	9.76	5.80	12.38
75	cupressene	1880	-	0.47	-
76	5-octen-2-one	1932			0.53
77	androsta-4,16-dien-3-one	1933	0.71	0.50	0.70
78	androsta-3,5-dien-7-one	1933	0.55	0.26	0.32
79	6-androstanone	1940			0.13
80	n-hexadecanoic acid	1977			0.43
81	9Z,12Z,15Z-octadecatrien-1-ol	2077	0.20		
82	pimara-7,15-dien-3-one	2097	0.36	0.16	0.24
83	thunbergol	2211	-	0.98	0.23
84	andrographolide	2944		0.84	0.74
**Total**			**96.55**	**94.56**	**95.35**

S.N. = Serial number.:, KI/RI= Kovat indicest/retention indices.

**Table 2:** Antioxidant properties in terms of IC_50_ values in essential oils from different areal parts of *C. macrophylla.*

Extracts	(IC _50_ in (µL/µg)/mL)/R^2^										% Absorbance (Reducing Power)	
	Radical scavenging Activities								Chelating Activity		At lower Dose Level (5 µL/µg)/mL	At Higher Dose Level (25 µL/µg)/mL
	DPPH Scavenging		NO scavenging		Super Oxide Scavenging		OH Scavenging					
	IC_50_	*R*^2^	IC_50_	*R*^2^	IC_50_	*R*^2^	IC_50_	*R*^2^	IC_50_	*R*^2^		
**CMLEO**	18.35 ± 0.18	0.933 ± 0.003	10.61 ± 0.02	0.941 ± 0.000	18.58 ± 0.19	0.956 ± 0.006	16.06 ± 0.16	0.963 ± 0.006	14.38 ± 0.27	0.913 ± 0.009	0.426 ± 0.004	0.602 ± 0.000
**CMEO-I**	20.29 ± .11	0.960 ± 0.06	11.18 ± 0.06	0.95 ± 0.004	20.79 ± 0.30	0.954 ± 0.005	18.59 ± 0.25	0.960 ± 0.006	13.42 ± 0.17	0.950 ± 0.004	0.411 ± 0.003	0.573 ± 0.000
**CMEO-II**	15.66 ± 0.03	0.961 ± 00.005	**7.37 ± 0.11**	0.922 ± 0.002	17.49 ± 0.13	0.954 ± 0.015	14. 59 ± 0.18	0.956 ± 0.016	11.49 ± 0.87	0.979 ± 0.006	0.450 ± 0.004	0.418 ± 0.000
**BHT**	8.55 ± 0.10	0.947 ± 0.005	NA	NA	NA	NA	NA	NA	NA	NA	0.55 ± 0.008	0.735 ± 0.009
**Catechin**	8.18 ± 0.11	0.950 ± 0.004	NA	NA	NA	NA	NA	NA	NA	NA	0.455 ± 0.006	0.623 ± 0.004
**Gallic Acid**	7.95 ± 0.11	0.964 ± 0.004	NA	NA	NA	NA	NA	NA	NA	NA	0.575 ± 0.003	0.715 ± 0.003
**Ascorbic acid**	NA	NA	7.72 ± 0.19	0.942 ± 0.002	15.03 ± 0.13	0.951 ± 0.007	11.22 ± 0.30	0.960 ± 0.017	NA	NA	NA	NA
**EDTA**	NA	NA	NA	NA	NA	NA	NA	NA	9.27 ± 0.11	0.955 ± 0.003	NA	NA
**Citric Acid**	NA	NA	NA	NA	NA	NA	NA	NA	9.42 ± 0.95	0.981 ± 0.021	NA	NA

*R*^2^ = linear regression factor, NA = not applicable.

**Table 3:** Effect of essential oils from *C. macrophylla* on carrageenan-induced paws edema.

Group	Treatment	Doses (0.2 mL)	Paw Volume (in mm^3^)			% Inhibition	
			0 h	4 h	24 h	4 h	24 h
I	CMLEO	5%	2.34 ± 0.01	2.29 ± 0.02^b^	2.23 ± 0.02 ^ab^	2.14	4.7
II	CMLEO	10%	2.31 ± 0.03 ^a^	2.21 ± 0.02 ^ab^	2.12 ± 0.01 ^ab^	4.33	8.23
III	CMLEO	20%	2.25 ± 0.02 ^ab^	2.05 ± 0.02 ^ab^	1.85 ± 0.03 ^ab^	8.89	17.78
IV	CMEO-II	5%	2.29 ± 0.01 ^a^	2.19 ± 0.02 ^ab^	2.14 ± 0.03 ^ab^	4.37	6.55
V	CMEO-II	10%	2.38 ± 0.02	2.30 ± 0.02 ^b^	2.21 ± 0.02 ^ab^	3.36	7.14
VI	CMEO-II	20%	2.41 ± 0.02	2.17 ± 0.02 ^ab^	1.90 ± 0.05 ^ab^	9.96	21.16
VII	CMEO-I	5%	2.32 ± 0.03 ^a^	2.29 ± 0.03 ^b^	2.21 ± 0.02 ^ab^	1.29	4.74
VIII	CMEO-I	10%	2.26 ± 0.02 ^ab^	2.20 ± 0.02 ^ab^	2.15 ± 0.03 ^ab^	2.65	4.87
IX	CMEO-I	20%	2.27 ± 0.02 ^a^	2.08 ± 0.03a ^b^	1.90 ± 0.03 ^ab^	8.37	16.3
X	Control	-	2.40 ± 0.02	2.33 ± 0.01	2.32 ± 0.01	2.92	3.33
XI	Ibuprofen	40 mg/kg b. wt.	2.34 ± 0.01 ^a^	1.73 ± 0.02 ^a^	1.47 ± 0.02 ^a^	26.07	37.17

^a^ = Significant (*p* < 0.05) as compared to control, ^b^ = Significant (*p* < 0.05) as compared to Ibuprofen. % = Percent reduction in paw volume at different times.

**Table 4:** Effect of essential oils from *C. macrophylla* on formalin-induced mice paw edema.

Group	Treatment	Dose	Volume of Paw Edema (in mm^3^)										
			0 Day	1 Day	2 Day	3 Day	4 Day	5 Day	6 Day	7 Day	8 Day	9 Day	10 Day
I	CMLEO	5% (0.2 mL)	2.28 ± 0.02	2.36 ± 0.02 ^ab^	2.37 ± 0.04 ^b^	2.42 ± 0.04 ^b^	2.41 ±0.04 ^b^	2.40 ± 0.04 ^b^	2.40 ± 0.04 ^b^	2.39 ± 0.04 ^b^	2.39 ± 0.03 ^b^	2.36 ± 0.03 ^b^	2.36 ± 0.04 ^b^
II	CMLEO	10% (0.2 mL)	2.23 ± 0.03	2.26 ± 0.02 ^ab^	2.31 ± 0.03 ^b^	2.35 ± 0.04 ^a^	2.34 ±0.03 ^b^	2.32 ± 0.03 ^b^	2.32 ± 0.03 ^b^	2.31 ± 0.03 ^b^	2.31 ± 0.03 ^b^	2.31 ± 0.03 ^b^	2.30 ± 0.03 ^b^
III	CMLEO	20% (0.2 mL)	2.23 ± 0. 02	2.25 ± 0.02 ^ab^	2.29 ± 0.02 ^b^	2.30 ± 0.04 ^a^	2.29 ± 0.04 ^ab^	2.27 ± 0.03 ^a^	2.26 ± 0.03 ^ab^	2.25 ± 0. 02 ^ab^	2.24 ± 0.02 ^a^	2.22 ± 0.02 ^a^	2.22 ± 0.02 ^b^
IV	CMEO -II	5% (0.2 mL)	2.27 ± 0.03	2.30 ± 0.02 ^ab^	2.36 ± 0.04 ^b^	2.40 ± 0.05 ^ab^	2.39 ± 0.05 ^b^	2.38 ± 0.05 ^b^	2.38 ± 0.04 ^b^	2.38 ± 0.03 ^b^	2.37 ± 0.03 ^b^	2.36 ± 0.03 ^b^	2.36 ± 0.03 ^a^
V	CMEO-II	10% (0.2 mL)	2.24 ± 0.03	2.27 ± 0.02 ^ab^	2.34 ± 0.04 ^b^	2.38 ± 0.04 ^ab^	2.37 ± 0.04 ^b^	2.36 ± 0.03 ^b^	2.35 ± 0.03 ^b^	2.33 ± 0.03 ^b^	2.32 ± 0.02 ^b^	2.31 ± 0.02 ^b^	2.30 ± 0.02 ^b^
VI	CMEO-II	20% (0.2 mL)	2.23 ± 0.02	2.24 ± 0.02 ^ab^	2.26 ± 0.02 ^a^	2.28 ± 0.03 ^a^	2.26 ± 0.03 ^a^	2.25 ± 0.02 ^a^	2.24 ± 0.02 ^ab^	2.24 ± 0.02 ^ab^	2.22 ± 0.03 ^a^	2.21 ± 0.02 ^a^	2.20 ± 0.03 ^a^
VII	CMEO-I	5% (0.2 mL)	2.28 ± 0.02	2.29 ± 0.02 ^ab^	2.37 ± 0.03 ^b^	2.42 ± 0.04 ^b^	2.41 ± 0.03 ^b^	2.41 ± 0.03 ^b^	2.41 ± 0.03 ^b^	2.41 ± 0.02 ^b^	2.41 ± 0.02 ^b^	2.41 ± 0.02 ^b^	2.40 ± 0.02 ^b^
VIII	CMEO-I	10% (0.2 mL)	2.26 ± 0.03	2.29 ± 0.02 ^ab^	2.32 ± 0.03 ^b^	2.36 ± 0.04 ^a^	2.35 ± 0.03 ^b^	2.35 ±0.03 ^b^	2.34 ± 0.02 ^b^	2.33 ± 0.03 ^b^	2.35 ± 0.03 ^b^	2.33 ± 0.03 ^b^	2.33 ± 0.03 ^b^
IX	CMEO-I	20% (0.2 mL)	2.26 ± 0.02	2.27 ± 0.02 ^ab^	2.29 ± 0.03 ^b^	2.31 ± 0.03 ^a^	2.30 ± 0.02 ^ab^	2.29 ± 0.02 ^ab^	2.27 ± 0.01 ^ab^	2.27 ± 0.02 ^ab^	2.25 ± 0.02 ^ab^	2.24 ± 0.02 ^a^	2.24 ± 0.02 ^ab^
X	Control	(0.2 mL)	2.13 ± 0.02	2.17 ± 0.04	2.38 ± 0.03	2.52 ± 0.04	2.44 ± 0.02	2.39 ± 0.01	2.38 ± 0.02	2.38 ± 0.01	2.36 ± 0.01	2.36 ± 0.02	2.37 ± 0.03
XI	Ibuprofen	10 mg/kg	2.11 ± 0.02	2.13 ± 0.02 ^a^	2.19 ±0.01 ^a^	2.27 ± 0.01 ^a^	2.21 ± 0.02 ^a^	2.19 ± 0.01 ^a^	2.15 ± 0.01 ^a^	2.16 ± 0.01 ^a^	2.17 ± 0.01 ^a^	2.19 ± 0.01 ^b^	2.15 ± 0.02 ^a^

^a^ = Significant (*p* < 0.05) as compared to control, ^b^ = Significant (*p* < 0.05) as compared to Ibuprofen.

**Table 5:** Effect of essential oils from *C. macophylla* on acetic acid induced writhing in mice.

Group	Treatment	Doses	Writhing Counts	% Writhings	Inhibition (%)
I	CMLEO	5%	115.83 ± 8.06 ^ab^	83.53	16.47
II	CMLEO	10%	107.00 ± 4.52 ^ab^	77.16	22.84
III	CMLEO	20%	95.00 ± 9.01 ^ab^	69.95	31.49
IV	CMEO -II	5%	112.33 ± 5.28 ^ab^	81	18.99
V	CMEO-II	10%	104.33 ± 7.09 ^ab^	75.24	24.76
VI	CMEO-II	20%	85.33 ± 7.74 ^a^	61.53	38.46
VII	CMEO-I	5%	119.67 ± 7.09 ^ab^	86.3	13.7
VIII	CMEO-I	10%	111.67 ± 6.12 ^ab^	80.53	19.47
IX	CMEO-I	20%	103.00 ± 4.86 ^ab^	74.28	25.72
X	Control	-	138.67 ± 5.75	-	-
XI	Ibuprofen	40 mg/kg b. wt.	77.67 ± 6.86 ^a^	56.01	43.99

^a^ = Significant (*p* < 0.05) as compared to control, ^b^ = Significant (*p* < 0.05) as compared to Ibuprofen.

**Table 6:** Effect of essential oils from *C. macrophylla* on brewer’s yeast-induced pyrexia in mice.

Group	Treatment	Dose	Body Temperature (°C)		Body Temperature After Administration of Drug (°C)			% Reduction in Body Temperature		
			Before Injection of Yeast	After 18 h of Yeast Injection	1 h	3 h	24 h	1 h	3 h	24 h
I	CMLEO	5% (0.2 mL)	37.57 ± 0.05	38.57 ± 0.04	38.29 ± 0.03 ^b^	38.18 ± 0.04 ^ab^	38.13 ± 0.03 ^b^	28	39	44.00
II	CMLEO	10% (0.2 mL)	37.47 ± 0.03	38.49 ± 0.03 ^b^	38.15 ± 0.03 ^ab^	38.01 ± 0.04 ^ab^	37.94 ± 0.04 ^ab^	33.33	47.05	53.92
III	CMLEO	20% (0.2 mL)	37.56 ± 0.02 ^a^	38.58 ± 0.04	37.99 ± 0.03 ^ab^	37.86 ± 0.04 ^ab^	37.77 ± 0.05 ^ab^	57.84	70.59	79.41
IV	CMEO-II	5% (0.2 mL)	37.55 ± 0.03	38.56 ± 0.03	38.27 ± 0.02 ^b^	38.12 ± 0.03 ^ab^	38.08 ± 0.03 ^ab^	28.71	43.56	47.52
V	CMEO-II	10% (0.2 mL)	37.49 ± 0.04	38.51 ± 0.03	38.14 ± 0.04 ^ab^	37.97 ± 0.04 ^ab^	37.90 ± 0.04 ^ab^	36.27	52.94	59.8
VI	CMEO-II	20% (0.2 mL)	37.51 ± 0.04	38.55 ± 0.03	37.89 ± 0.04 ^ab^	37.73 ± 0.02 ^ab^	37.63 ± 0.03 ^a^	63.46	78.85	88.46
VII	CMEO-I	5% (0.2 mL)	37.48 ± 0.03	38.46 ± 0.03 ^b^	38.19 ± 0.03 ^ab^	38.12 ± 0.04 ^ab^	38.06 ± 0.04 ^ab^	27.55	34.69	40.82
VIII	CMEO-I	10% (0.2 mL)	37.52 ± 0.02	38.53 ± 0.03	38.20 ± 0.04 ^ab^	38.07 ± 0.04 ^ab^	38.00 ± 0.05 ^ab^	32.67	45.54	52.47
IX	CMEO-I	20% (0.2 mL)	37.49 ± 0.02	38.54 ± 0.02	37.97 ± 0.02 ^ab^	37.87 ± 0.02 ^ab^	37.76 ± 0.05 ^ab^	54.28	63.81	74.29
X	Control	(0.2 mL)	37.46 ± 0.02	38.50 ± 0.03	38.36 ± 0.04	38.30 ± 0.03	38.22 ± 0.03	13.46	19.23	26.92
XI	Paracetamol	33.0 mg/kg	37.50 ± 0.03	38.59 ± 0.04 ^a^	37.75 ± 0.04 ^a^	37.61 ± 0.03 ^a^	37.54 ± 0.04 ^a^	77.06	89.91	96.33

^a^ = Significant (*p* < 0.05) as compared to control, ^b^ = Significant (*p* < 0.05) as compared to Paracetamol.
